# The Effects of Exercise on Coronary Collateral Circulation: A Review

**DOI:** 10.7759/cureus.32732

**Published:** 2022-12-20

**Authors:** Robert Ambrogetti

**Affiliations:** 1 Medicine, Peterborough City Hospital, Peterborough, GBR

**Keywords:** collateral, resistance training, preoperative exercise therapy, exercise training, physical acitivity, coronary collateral circulation

## Abstract

The effects of exercise on the cardiovascular system are multifaceted and complex. It is well-documented that exercise can reduce mortality related to cardiovascular pathology. One anatomical structure that has been implicated in this process is the coronary collateral circulation. The goal of this review is to evaluate the current literature on the effects of exercise on human coronary collateral circulation.

A search for literature was conducted on the databases Science Direct and PubMed using the terms: coronary collateral, collateral, exercise, physical activity, resistance training, endurance training, and collateral artery. Research that had the primary outcome of assessing human coronary collateralization secondary to exercise was included. Research in which the effect of exercise was not the primary outcome was excluded. As a result, a total of 13 research papers on the effects of exercise on coronary collateral circulation were included.

Thirteen original research papers were reviewed. The mean age range in all studies was between 48 and 64 years old. There was a predominance of male participants, with a total of 597 male patients and 108 female patients across all studies. It was found that initial research underestimated the effect of exercise on coronary collateral circulation due to a lack of sensitive assessment methods. With the introduction of sensitive measures like the collateral flow index (CFI) and Rentrop scoring, results have shown that coronary collateral function can be increased with exercise.

Exercise has been shown to enhance coronary collateral function. There is limited evidence as to which type, duration, or intensity of exercise is most favourable to enhance coronary collateral function. There is also relatively little data on the effects of exercise in the female population and those over the age of 65 years. More research is required to determine the specific effects of exercise on coronary collateral circulation in various age groups, genders, co-morbidities, specific exercise modalities, durations, intensities, and the effect of pharmacotherapy.

## Introduction and background

The benefits of exercise on coronary circulation were first proposed by William Heberden in the mid-eighteenth century when he observed that a patient with angina had significantly improved symptoms after six months of daily physical activity [1]. The effects of exercise on coronary circulation have since been shown to be multifactorial, including improvement in coronary endothelial function, economisation of ventricular function, reduction in cardiovascular risk factors, and enhanced coronary collateral blood flow [2,3].

The importance of coronary collateral flow in reducing myocardial ischaemic insults and associated mortality in acute and chronic coronary artery disease (CAD) is well documented [4]. The presence of enhanced collateral circulation is associated with a reduction in the occurrence and extent of myocardial infarctions [5-7]. In a meta-analysis by Meier et al. (2012), "high collateralization" in the context of stable CAD was associated with a 36% reduction in mortality when compared to patients with "low collateralization" [8].

Human coronary circulation consists of an interconnected arterial network with an underlying system of connected collateral vessels [4]. This system of collateral vessels is present from birth, varies between individuals, and changes over the human lifespan [4]. Factors that have been shown to influence the function of coronary circulation include the enlargement of pre-existing collateral vessels (arteriogenesis), the formation of new collateral vessels (angiogenesis), age, genetic factors, and exercise [4,9]. Hence, the function of coronary collateral circulation can be altered by a complex interplay between multiple factors over time.

The primary mechanism that is thought to enhance coronary collateralization through exercise is increasing pressure gradients between proximal and distal arteries, which are already partially occluded [4,10]. This causes increased pressure of blood flow through collateral vessels as the blood moves from zones of higher pressure proximally to occluded vessels' lower pressure zones distally [4,10]. The resultant increased physical stimulus on collateral vessel walls triggers an adaptive cascade of cellular proliferation and structural remodelling, which has been reported to potentially increase collateral vessel diameter by up to 20-fold [10].

This review will evaluate the evidence in relation to the effect of exercise on coronary collateral circulation. Areas for potential further research will be highlighted.

## Review

Methods

A literature search of online databases (Science Direct and PubMed) was conducted, using the terms "coronary collateral," "collateral," "exercise," "physical activity," "resistance training," and "collateral artery." Further relevant research papers were found by reviewing the reference lists of key articles. Articles were initially reviewed by title, abstract, and then full text. Inclusion criteria included research papers that had primary outcomes assessing human coronary collateralization secondary to exercise. When exercise was not one of the main interventions, research papers were excluded. As a result, 13 research papers were included in this review. A lack of heterogeneity in study design, exercise protocols, and outcome measures precludes a meta-analysis.

Results 

Increased coronary collateralization secondary to vessel narrowing and augmentation of this process with exercise has been shown in studies on animals since 1957 [11]. Early clinical trials in humans before 1998 showed that exercise had beneficial effects on myocardial function, exercise capacity, ventricular function, and anginal symptoms [12-17]. However, all early observational studies failed to show that any of these benefits were related to exercise-induced improvements in the collateralization of coronary circulation [12-14]. The initial observational trial by Ferguson et al. (1974) failed to show any significant coronary collateralization over a 13-month period of exercise using angiography. This trial involved one hour per week of low-intensity exercise (e.g., walking and recreational volleyball) [13]. Two of the 14 patients in this study showed increased coronary collateralization on angiography; however, this was attributed to the progression of CAD to over 95% occlusion in both cases [13]. This study was likely further confounded by the small sample size, lack of attendance (68% +/- 16%), and a low-intensity exercise programme (e.g., walking and recreational games). Similar results were seen in an observational study by Conner et al. (1976). Over a 12-month period, 1 hour of exercise per week resulted in no significant increase in coronary collateralization between pre-and post-intervention angiography. Two of the patients in this trial were shown to have increased collateralization. However, like Fergusson et al. (1974), both patients were shown to have concurrent progression of their CAD to more than 90% occlusion. Hence, collateralization in these patients was attributed to increased vessel occlusion and not exercise [12]. This study was confounded by the low sample size, variable amounts of coronary stenosis at baseline, and lack of a control group.

Verani et al. [14] pioneered the use of thallium-201 exercise scintigraphy to assess the effects of collateralization secondary to exercise. In this observational study, 16 patients’ coronary collateral circulation was assessed using scintigraphy pre- and a 12-week exercise programme (one hour of exercise per week up to 80% of the maximum heart rate). Four patients showed increased myocardial perfusion, two showed reduced myocardial perfusion, and the others had no significant change. Overall, no significant change in myocardial perfusion at similar cardiac workloads pre- and post-intervention was noted, suggesting no significant change in coronary collateralization [14]. This study was potentially limited by the variable amount of coronary stenosis at baseline, the use of propranolol by nine patients, and the lack of sensitivity for collateral circulation with scintigraphy.

Nolewajka et al. randomly allocated 20 men with known CAD into an exercise intervention group or control group. Baseline angiography was performed in both groups prior to the intervention period. The exercise intervention consisted of five one-hour exercise sessions per week (four supervised and one unsupervised) at an intensity of up to 70% of the maximum heart rate. Post-intervention angiography at seven months showed no difference in collateral development compared to controls [17]. The exercise group was noted to have an increased angina threshold post-intervention. However, it was difficult to attribute this to coronary collateralization without objective evidence. It was also difficult to draw comparisons between the two groups as the control group also participated in group exercise sessions twice per week. It was not possible to confirm the compliance with exercise in the unsupervised sessions, although the magnitude to which this would affect results is uncertain.

A lack of collateral development on angiography was also noted in 133 patients with known CAD who underwent 3 weeks of daily inpatient exercise and education [16]. The exercise intervention involved 30 minutes per day at up to 75% of the maximum heart rate and a twice-weekly supervised group session. The intervention group also received education on lifestyle and diet. This was compared to a control group that only received the same education on diet and exercise. This study was confounded by overall low adherence to exercise in the intervention group. As adherence was reported to be around 68% for group sessions and around 60% for home exercise, the control group was given the same exercise advice as the intervention group. It is not possible to know the intensity or amount of exercise completed in the control group. Hence, drawing conclusions around differences or lack of differences between intervention and control is difficult. 

All the aforementioned studies had multiple limitations [12-17]. Other than unsupervised exercise, a lack of control groups, and generally low-intensity exercise programs, these studies all employed standard angiography to assess for changes in coronary collateralization. The size of coronary collateral vessels is between 30 and 200 μm which is below what digital angiographic imaging systems can reliably detect [4]. Hence, it is likely that these early studies would have underestimated changes in coronary collateral circulation in response to exercise. It should be noted that some early studies tried to use cardiac scintigraphy to measure coronary collateral circulation [14,16,17]. Changes observed in scintigraphy were difficult to attribute solely to increased coronary collateralization as other factors like improvement in coronary vessel resistance may have been responsible [4].

In 1998, two studies utilising the collateral flow index (CFI) and Rentrop scoring (angiographic scoring systems) were able to demonstrate improved coronary collateralization secondary to exercise [18, 19]. Belardinelli et al. (1998) used the angiographic scoring from Rentrop et al. (1985) to show coronary collateralization significantly increased after two months of exercise intervention. In this randomised control trial (RCT), 50 patients with known CAD were divided into an exercise intervention group and a control group that avoided exercise. The exercise intervention consisted of eight weeks of one hour of moderate-intensity exercise (60% of the predetermined Vo2 max) on a cycle ergometer three times per week. Baseline and post-intervention angiography, cardiopulmonary exercise testing (CPET), and stress echocardiography were compared in both groups. In comparison to the control group, the intervention group had an improved collateral circulation score (p = < 0.001), myocardial perfusion (p = < 0.001), and ventricular function (p = <0.001) in the absence of progression of coronary stenosis. Senti et al. (1998) developed a coronary collateral flow index (CFI) using angiography. In this study, 79 patients with CAD completed standardised interviews to determine their amount of physical activity during work and leisure. This cohort cross-sectional study showed that higher physical activity during leisure time was associated with increased CFI at angiography in patients with CAD. Limitations to this study include the potential for inaccurate recounts of physical activity and the lack of an objective measure to quantify physical activity.

Following this, multiple RCTs have demonstrated increased CFI in response to exercise [3,20-23]. The CFI has also been used to show increased collateral function in response to different types of exercise (endurance, isometric, supine bike, and isolated resistance training) and intensities (60%-95% of pre-determined maximums) [3,20-23].

Endurance training at moderate and high intensities has been shown to increase coronary collateralization in multiple studies [3,21]. In a retrospective cohort study by Zbinden et al. (2006), 40 patients with CAD were assessed after three months of supervised cardiac rehabilitation. This included jogging and walking three times per week for one hour at 80% of the predetermined maximum intensity (as determined at cardiopulmonary exercise testing (CPET)). Patients were divided into an exercising group and a sedentary group retrospectively based on their adherence to the cardiac rehabilitation program. Collateral flow index (CFI) measurements were performed at baseline and post-intervention. The patients in the exercise group showed an increase in coronary collateral function in normal and stenotic vessels compared to the non-exercising group (p = < 0.03). A direct correlation between enhancement in Vo2 max and increased coronary collateralization (p = <0.007) was seen. An RCT trial by Mobius et al. (2016) supports these findings. In this study, 60 patients were randomly assigned to receive supervised high-intensity exercise, supervised moderate-intensity exercise, or regular unsupervised exercise. The high-intensity group performed four 30-minute sessions a day, five times per week, at an intensity between 70 and 90% of the predetermined maximum angina threshold. The moderate-intensity group underwent six to eight sessions of 20 minutes each, five times per week, at 60% of their predetermined angina threshold. The control group was encouraged to perform regular physical activity two to three times per week for 20 to 30 minutes. CFI was used as the primary endpoint for pre- and post-angiography. There was a significant increase in CFI in the high-intensity (p = < 0.005) and moderate-intensity (p = < 0.004) groups compared to the control group, which had no observable increase in CFI. Increases in CFI were also found to be associated with higher ischaemic thresholds in the high and moderate-intensity exercise groups (p = 0.006). One of the main limitations of this study is the unrealistic nature of the exercise regime. It would likely be impractical for the general population to complete three to five sessions of exercise per day consistently.

Exercise over short durations (one to six minutes) has been shown to acutely increase CFI [22, 24, 25]. In an RCT by Pohl et al. (2003), dynamic handgrip exercise at 60% of maximum for two minutes during angiography was shown to increase CFI two-fold, compared to the control group with no hand grip exercises (p = <0.0001). Interestingly, patients on beta blockers did not show a significant increase in CFI during hand grip exercises. Similar findings were shown in an RCT by Lin et al. (2012). In this study, 65 patients with CAD were randomly assigned to one minute of isometric handgrip exercise or no exercise during angiography. The isometric hand group showed a significant increase in CFI compared to the control (p=<0.01). Systolic and diastolic blood pressures were also noted to be increased compared to controls (p = < 0.01). Togni et al. (2010) supported their findings with a randomised cross-over trial. Togni et al. measured CFI at rest and then during the last one minute of a six-minute period of exercise on a supine bicycle ergometer. There was a statistically significant increase in CFI during exercise compared to resting measurements (p = 0.0002).

Several studies have assessed the potential for medications to augment the effects of exercise on coronary collateralization [15,24,25]. The effects of concomitant exercise and medication on enhancing coronary collateralization were first studied by Fujita et al. in 1988. In this study, 16 patients with CAD were randomly divided into two groups. One group had 5000 units of heparin administered intravenously before exercise, while the control group performed exercise alone. This consisted of treadmill-based exercise as per the Bruce protocol [26] at an intensity of 80% of the predetermined angina threshold. This was performed twice a day for 10 days in both groups. Changes in collateralization were assessed by pre- and post-intervention angiography. The exercise group with pre-treatment heparin was shown to have significantly increased collateralization compared to the baseline (p = <0.005). It was difficult to compare between groups as the exercise-only group did not have a repeat assessment of coronary collateral function post-intervention. The effect of exercise in conjunction with heparin on coronary collateral function was further assessed in a double-blind RCT by Petrovic et al. in 2021. In this study, 32 patients with known CAD were randomly assigned to one of two groups: exercise with intravenous heparin (at 100 units per kg; maximum dose of 5000 IU) or exercise alone. CT angiography was used to assess the coronary circulation pre- and post-intervention, using the Rentrop classification [27]. Similar to Fujita et al. (1988), both groups performed 10 days of treadmill exercise as per the Bruce protocol [26]. The exercise with the heparin group had significantly increased coronary collateral function compared to baseline (p = <0.001). The coronary collateral function remained unchanged in the exercise-only group. The use of CT coronary angiography in this study has the benefit of being less invasive than standard coronary angiography. However, it lacks the sensitivity to detect changes in the coronary collateral circulation when compared to the CFI used in previous studies [4]. Hence, the use of a CT coronary angiogram in this study may have underestimated the effect on coronary collateralization in both the control and intervention groups. Most patients in clinical settings receive heparin subcutaneously. Hence the results of both the above studies [15,28] may not translate into the clinical setting. These two studies had relatively small sample sizes and short durations of exercise and medication. Hence, it should be used only for hypothesis generation. 

Discussion, limitations, and future research 

The main driver for increased coronary collateral development is a change in coronary vessel pressures, which results in an increased shear force on collateral vessel walls [4]. One way in which the shear force on coronary vessel walls is increased is with exercise [4]. Initial research failed to show any significant effect of exercise on coronary collateralization [12-17]. This was most likely due to the lack of sensitivity of standard angiography in the assessment of coronary collaterals. The advancement of technology and the development of the CFI (gold standard) and Rentrop scoring systems allowed for more sensitive measurement of coronary collateral circulation in response to exercise [4,19]. As a result, exercise over different modalities (endurance, isometric, isolated resistance), durations (one minute-12 months), and intensities (60%-90%) has since been shown to have a positive effect on coronary collateralization [3,18-23].

Most evidence for the positive effects of exercise on coronary collateralization has been shown in short-term (one month) and long-term (12 months) responses to endurance-type exercises at moderate to high intensities [3,18,19,21]. Interestingly, the moderate intensity (60% of angina-free maximum) group in Mobius et al. (2016) showed higher increases in coronary collateral function than the high intensity (70%-95% of angina-free maximum) group [3]. Given that coronary circulation is a purely diastolic phenomenon, this may reflect lower heart rates in the moderate-intensity group [4]. However, this highlights the need for further research into which intensities of endurance exercise are optimal for enhancing coronary collateral function. No study has assessed the effects of exercise on the coronary collateral system beyond a period of one year. Hence, the long-term effects of exercise on coronary collateralization beyond one year remain to be assessed.

Acute effects during short bursts of isometric and isolated resistance training lasting one to two minutes have also been shown to increase coronary collateral function [20,23]. These studies have highlighted the possible effect of changes in haemodynamics (mainly elevations in blood pressure) with resistance exercise and CFI. Only one study has assessed the effects of isolated resistance exercise on coronary collateral circulation [20]. This study found that isolated hand exercises for one minute significantly increased coronary collateralization [20]. This study highlighted the need for further research into the effects of resistance training, both in terms of isolated and compound movements. To date, there are no studies on the effects of long-term resistance training on coronary collateral function.

Clinical trials evaluating the effects of exercise on coronary collateralization have invariably involved patients with pre-existing CAD. This is due to several reasons. Patients with CAD are a population that stands to benefit from increased coronary collateralization [8,18,29]. Many of these patients will be undergoing angiography as part of their diagnostic workup and treatment. Hence, they are more likely to volunteer and be accessible for studies involving angiography, whereas healthy patients may be less likely to volunteer due to the lack of sensitive tests with low risk-to-benefit profiles. As all studies involve only patients with CAD, there is no evidence for coronary collateralization in response to exercise in healthy patients. As expected, with the study population being only CAD patients, there is a gender bias toward the selection of males. There were 597 male patients and 108 female patients in all studies reviewed, limiting data on the effects in females. Further research into the different effects of exercise on coronary collateral circulation specifically in women and the differences between genders is required.

Generally, CAD is more prevalent and more severe in patients over the age of 65 [30]. The mean age range across all studies was between 48 and 64 years old. This is due to the fact that most older patients are excluded due to a higher prevalence of co-morbidities, leaving little data in older age groups. However, if exercise can increase coronary collateral function in patients over 65, they may stand to benefit the most from such interventions. Therefore, further research is required on the effects of exercise on coronary collateralization in older populations.

Exercise in conjunction with heparin has been shown to increase collateralization [15,24]. These findings suggest that there is a potential role for pharmacotherapy in conjunction with exercise to enhance coronary collateralization. However, no studies have used the gold standard CFI to evaluate the effects to date. In contrast, some medications used in CAD, like beta blockers, may have a negative effect on exercise-induced coronary collateralization [9,20]. As most patients with CAD and the ageing population are on multiple medications, further research on the effects of pharmacotherapy and exercise on coronary collateralization will help guide management.

The use of a generalised "exercise load" parameter (such as heart rate times by duration of exercise) to quantify exercise and compare this to changes in CFI may allow for easier application in a clinical setting. The use of an "exercise load" may allow for a dose-response relationship between exercise and CFI to be measured. This may provide insights into the optimal dosing of exercise. An exercise loading concept may also increase patient autonomy by allowing patients to pick and choose the exercise modalities and durations that they prefer. The use of exercise loads in the assessment of coronary collateralization may also promote integration with technology (e.g., computer software, monitoring equipment, and artificial intelligence). Both the potential for integration with technology and increased patient autonomy could increase long-term adherence to exercise programmes and provide more robust data for analysis in the long term.

## Conclusions

Exercise has been shown to enhance coronary collateral function. However, drawing firm conclusions that will guide recommendations from the current evidence is difficult due to the heterogeneity of study design, durations, and exercise protocols used. Further research into the specific effects of exercise on coronary collateral circulation in different age groups, gender, co-morbidities, specific exercise modalities, durations, intensities, and the effect of pharmacotherapy is still required. 
